# Potential biological weapons threats.

**DOI:** 10.3201/eid0504.990411

**Published:** 1999

**Authors:** M G Kortepeter, G W Parker

**Affiliations:** U.S. Army Medical Research Institute of Infectious Diseases, Fort Detrick, Maryland 21702-5011, USA. Mark_Kortepeter@DET.AMEDD.ARMY.MIL


### Potential Biological Weapons Threats

**Mark G. Kortepeter and Gerald W. Parker**
U.S. Army Medical Research Institute of Infectious Diseases,
Fort Detrick, Maryland, USA

The list of agents that could pose the greatest public health risk in the event of a bioterrorist attack is short. However, although short, the list includes agents that, if acquired and properly disseminated, could cause a difficult public health challenge in terms of our ability to limit the numbers of casualties and control the damage to our cities and nation.

The use of biological weapons has occurred sporadically for centuries, culminating in sophisticated research and testing programs run by several countries. Biological weapons proliferation is a serious problem that is increasing the probability of a serious bioterrorism incident. The accidental release of anthrax from a military testing facility in the former Soviet Union in 1979 and Iraq's admission in 1995 to having quantities of anthrax, botulinum toxin, and aflatoxin ready to use as weapons have clearly shown that research in the offensive use of biological agents continued, despite the 1972 Biological Weapons Convention (1,2). Of the seven countries listed by the U.S. Department of State as sponsoring international terrorism (3), at least five are suspected to have biological warfare programs. There is no evidence at this time, however, that any state has provided biological weapons expertise to a terrorist organization (4).

A wide range of groups or individuals might use biological agents as instruments of terror. At the most dangerous end of the spectrum are large organizations that are well-funded and possibly state-supported. They would be expected to cause the greatest harm, because of their access to scientific expertise, biological agents, and most importantly, dissemination technology, including the capability to produce refined dry agent, deliverable in milled particles of the proper size for aerosol dissemination. The Aum Shinrikyo in Japan is an example of a well-financed organization that was attempting to develop biological weapons capability. However, they were not successful in their multiple attempts to release anthrax and botulinum toxin (4). On this end of the spectrum, the list of biological agents available to cause mass casualties is small and would probably include one of the classic biological agents. The probability of occurrence is low; however, the consequences of a possible successful attack are serious.

Smaller, less sophisticated organizations may or may not have the intent to kill but may use biological pathogens to further their specific goals. The Rajhneeshees, who attempted to influence local elections in The Dalles, Oregon, by contaminating salad bars with *Salmonella* Typhimurium, are an example (5). Rather than having a sophisticated research program, these organizations could use biological pathogens that are readily available.

The third type are smaller groups or individuals who may have very limited targets (e.g., individuals or buildings) and are using biological pathogens in murder plots or to threaten havoc. The recent anthrax hoaxes are examples of this. Many biological agents could be used in such instances and the likelihood of their occurrence is high, but the public health consequences are low.

There are many potential human biological pathogens. A North Atlantic Treaty Organization handbook dealing with biological warfare defense lists 39 agents, including bacteria, viruses, rickettsiae, and toxins, that could be used as biological weapons (6). Examining the relationship between aerosol infectivity and toxicity versus quantity of agent illustrates the requirements for producing equivalent effects and narrows the spectrum of possible agents that could be used to cause large numbers of casualities. For example, the amount of agent needed to cover a 100-km$^2$ area and cause 50%

Address for correspondence: Mark G. Kortepeter, Operational Medicine Division, U.S. Army Medical Research Institute of Infectious Diseases, Fort Detrick, MD 21702-5011, USA; fax: 301-619-2312; e-mail: Mark_Kortepeter@DET.AMEDD.ARMY.MIL.

lethality is 8 metric tons for even a "highly toxic" toxin such as ricin versus only kilogram quantities of anthrax needed to achieve the same coverage. Thus, deploying an agent such as ricin over a wide area, although possible, becomes impractical from a logistics standpoint, even for a well-funded organization (7). The potential impact on a city can be estimated by looking at the effectiveness of an aerosol in producing downwind casualties. The World Health Organization in 1970 modeled the results of a hypothetical dissemination of 50 kg of agent along a 2-km line upwind of a large population center. Anthrax and tularemia are predicted to cause the highest number of dead and incapacitated, as well as the greatest downwind spread (8).

For further indication of which pathogens make effective biological weapons, one could look at the agents studied by the United States when it had an offensive biological weapons research program. Under that program, which was discontinued in 1969, the United States produced the following to fill munitions: *Bacillus anthracis,* botulinum toxin, *Francisella tularensis, Brucella suis*, Venezuelan equine encephalitis virus, staphylococcal enterotoxin B, and *Coxiella burnetti* (9). As a further indication of which pathogens have the requisite physical characteristics to make good biological weapons, one need only look next at the agents that former Soviet Union biological weapons experts considered likely candidates. The agents included smallpox, plague, anthrax, botulinum toxin, equine encephalitis viruses, tularemia, Q fever, Marburg, melioidosis, and typhus (10,11). Criteria such as infectivity and toxicity, environmental stability, ease of large-scale production, and disease severity were used in determining which agents had a high probability of use. Both the United States before 1969 and the former Soviet Union spent years determining which pathogens had strategic and tactical capability.

The National Defense University recently compiled a study of more than 100 confirmed incidents of illicit use of biological agents during this century (W.S. Carus, pers. comm. [4]). Of the 100 incidents, 29 involved agent acquisition, and of the 29, 19 involved the actual nongovernmental use of an agent, and most were used for biocrimes, rather than for bioterrorism. In the context of this study, the distinguishing feature of bioterrorism is that it involves the use of "violence on behalf of a political, religious, ecologic, or other ideologic cause without reference to the moral or political justice of the cause." The balance of incidents involved an expressed interest, threat of use, or an attempt to acquire an agent. In the 1990s, incidents increased markedly, but most have been hoaxes. The pathogens involved present a wide spectrum, from those with little ability to cause disease or disability, such as *Ascaris suum*, to some of the familiar agents deemed most deadly, such as *B. anthracis*, ricin, plague, and botulinum toxins (Table). During this period, the number of known deaths is only 10, while the total number of casualties is 990. However, the numbers should not give a false sense of security that mass lethality is not achievable by a determined terrorist group. The sharp increase in biological threats, hoaxes, information, and Internet sources on this subject seen in recent years indicates a growing interest in the possible use of biological pathogens for nefarious means (4).

In general, the existing public health systems should be able to handle most attempts to release biological pathogens. A working group organized by the Johns Hopkins Center for Civilian Biodefense Studies recently looked at potential biological agents to decide which present the greatest risk for a maximum credible event from a public health perspective. A maximum credible event would be one that could cause large loss of life, in addition to disruption, panic, and overwhelming of the civilian health-care resources (12).

To be used for a maximum credible event, an agent must have some of the following properties: the agent should be highly lethal and easily produced in large quantities. Given that the aerosol route is the most likely for a large-scale attack, stability in aerosol and capability to be dispersed (1 µm to 5 µm particle size) are necessary. Additional attributes that make an agent even more dangerous include being communicable from person to person and having no treatment or vaccine.

When the potential agents are reviewed for these characteristics, anthrax and smallpox are the two with greatest potential for mass casualties and civil disruption. 1) Both are highly lethal: the death rate for anthrax if untreated before onset of serious symptoms exceeds 80%; 30% of unvaccinated patients infected with

Table 1. Biological agents involved in bioterrorism or biocrimes[a]

| | Traditional biological warfare agents | Agents associated with biocrimes and bioterrorism |
|---|---|---|
| Pathogens | *Bacillus anthracis*[b] | *Ascaris suum* |
| | *Brucella suis* | *Bacillus anthracis*[b] |
| | *Coxiella burnetii*[b] | *Coxiella burnetii*[b] |
| | *Francisella tularensis* | *Giardia lamblia* |
| | Smallpox | HIV |
| | Viral encephalitides | *Rickettsia prowazekii* (typhus) |
| | Viral hemorrhagic fevers[b] | *Salmonella* Typhimurium |
| | *Yersinia pestis*[b] | *Salmonella typhi* |
| | | *Shigella* species |
| | | *Schistosoma* species |
| | | *Vibrio cholerae* |
| | | Viral hemorrhagic fevers (Ebola)[b] |
| | | Yellow fever virus |
| | | *Yersinia enterocolitica* |
| | | *Yersinia pestis*[b] |
| Toxins | Botulinum[b] | Botulinum[b] |
| | Ricin[b] | Cholera endotoxin |
| | Staphylococcal enterotoxin B | Diphtheria toxin |
| | | Nicotine |
| | | Ricin[b] |
| | | Snake toxin |
| | | Tetrodotoxin |
| Anti-crop agents | Rice blast | |
| | Rye stem rust | |
| | Wheat stem rust | |

[a]Includes agents which were used, acquired, attempted to acquire, involved in a threat of use or an expressed interest in using. Reprinted with permission from Carus WS. Table 6: Biological agents involved. In: Carus WS. Bioterrorism and biocrimes: the illicit use of biological agents in the 20th Century. Working Paper, Center for Counterproliferation Research, National Defense University. August 1998, revised March 1999.
[b]These agents appear on both lists.

variola major could die. 2) Both are stable for transmission in aerosol and capable of large-scale production. Anthrax spores have been known to survive for decades under the right conditions (13). WHO was concerned that smallpox might be freeze-dried to retain virulence for prolonged periods (8). 3) Both have been developed as agents in state programs. Iraq has produced anthrax for use in Scud missiles and conducted research on camelpox virus, which is closely related to smallpox (2). A Soviet defector has reported that the former Soviet Union produced smallpox virus by the ton (11). 4) Use of either agent would have a devastating psychological effect on the target population, potentially causing widespread panic. This is in part due to the agents' well-demonstrated historical potential to cause large disease outbreaks (14). 5) Initial recognition of both diseases is likely to be delayed. For anthrax, this is secondary to the rare occurrence of inhalation anthrax. Only 11 cases of inhalation anthrax have been reported in the United States from 1945 to 1994 (15), and recognition may be delayed until after antibiotic use would be beneficial. For smallpox, given that few U.S. physicians have any clinical experience with the disease, many could confuse it for more common diseases (e.g., varicella and bullous erythema multiforme) early on, allowing for second-generation spread (12,16). 6) Availability of vaccines for either disease is limited. Anthrax vaccine, licensed in 1970, has been used for persons at high risk for contact with this disease. The U.S. military has recently begun vaccinating the entire force; however, there is limited availability of the vaccine for use in the civilian population. Routine smallpox vaccination was discontinued in the United States in 1971. Recent estimates of the current number of doses in storage at CDC range from 5 to 7 million (12), but the viability of stored vaccine is no longer guaranteed.

Obtaining smallpox virus as opposed to other agents (e.g., anthrax, plague, and botulinum toxin) would be difficult, but if obtained and intentionally released, smallpox could cause a public health catastrophe because of its communicability. Even a single case could lead to 10 to 20 others. It is estimated that no more than 20% of the population has any immunity from prior vaccination (12). There is no acceptable treatment, and the communicability by aerosol requires negative-pressure isolation. Therefore, these limited isolation resources in medical facilities would be easily overwhelmed.

Anthrax can have a delayed onset, further leading to delays in recognition and treatment. In the outbreak of inhalation anthrax in Sverdlovsk in 1979, some patients became ill up to 6 weeks after the suspected release of anthrax spores (1). The current recommendation for prophylaxis of persons exposed to aerosolized anthrax is treatment with antibiotics for 8 weeks in the absence of vaccine or 4 weeks and until three doses of vaccine have been given (17). The amount of antibiotics required for postexposure prophylaxis of large populations could be enormous and could easily tax logistics capabilities for consequence management.

Other bacterial agents capable of causing a maximum credible event include plague and tularemia. Plague, like smallpox and anthrax, can decimate a population (as in Europe in the Middle Ages). An outbreak of plague could easily cause great fear and hysteria in the target population (as in the 1994 outbreak in India), when hundreds of thousands were reported to have fled the city of Surat, various countries embargoed flights to and from India, and importation of Indian goods was restricted (18). Both plague and tularemia are potentially lethal without proper treatment; however, the availability of effective treatment and prophylaxis may reduce possible damage to a population. Both are infectious at low doses. Pneumonic plague's person-to-person communicability and untreated case-fatality rate of at least twice that of tularemia make it more effective than tularemia as an agent to cause mass illness.

Other agents of concern include the botulinum toxins and viral hemorrhagic fevers. Once again, both are highly lethal. Botulinum toxin is a commonly cited threat, and Iraq has admitted to producing it. Since intensive care would be required in treating both illnesses and ventilator management is life-saving for botulinum, both would easily tax existing medical care facilities. However, botulinum toxin may be a less effective agent because of relatively lower stability in the environment and smaller geographic coverage than other agents demonstrated in modeling studies. Producing and dispensing large amounts are also difficult (W.C. Patrick, pers. comm.,19).

A number of different viruses can cause hemorrhagic fever. These include (but are not limited to) Lassa fever, from the Arenaviridae family; Rift Valley fever and Crimean Congo hemorrhagic fever, from the Bunyaviridae family; and Ebola hemorrhagic fever and Marburg disease, from the Filoviridae family. These organisms are potential biological agents because of their lethality, high infectivity by the aerosol route shown in animal models, and possibility for replication in tissue culture (16).

In summary, we know that biological pathogens have been used for biological warfare and terrorism, and their potential for future use is a major concern. Therefore we must be prepared to respond appropriately if they are used again. The technology and intellectual capacity exist for a well-funded, highly motivated terrorist group to mount such an attack. Although the list of potential agents is long, only a handful of pathogens are thought to have the ability to cause a maximum credible event to paralyze a large city or region of the country, causing high numbers of deaths, wide-scale panic, and massive disruption of commerce. Diseases of antiquity (including anthrax, smallpox, and plague), notorious for causing large outbreaks, still head that list. In addition, other agents, such as botulinum toxin, hemorrhagic fever viruses, and tularemia, have potential to do the same. By focusing on a smaller list of these low-likelihood, but high-impact diseases, we can better prepare for potential intentional releases, and hope to mitigate their ultimate impact on our citizens.

Many other pathogens can cause illness and death, and the threat list will always be dynamic.

We must, therefore, have the appropriate surveillance system and laboratory capability to identify other pathogens, and we must improve our public health and medical capabilities to respond to the short list of the most dangerous naturally occurring biological pathogens that could be used as bioterrorism weapons.

Dr. Kortepeter is a preventive medicine officer in the Operational Medicine Division at the U.S. Army Medical Research Institute of Infectious Diseases, where he teaches the medical management of biological weapons casualties.

Dr. Parker is Commander, U.S. Army Medical Research Institute of Infectious Diseases (USAMRIID) at Fort Detrick, MD. USAMRIID conducts research to develop vaccines, medications, and diagnostics to protect U.S. service members from biological warfare threats and endemic infectious diseases.

### References

1. Meselson M, Gillemin J, Hugh-Jones M, Langmuir A, Popova I, Shelokov A, et al. The Sverdlovsk anthrax outbreak of 1979. Science 1994;209:1202-8.
2. Zilinskas RA. Iraq's biological weapons: the past as future? JAMA 1997;278:418-24.
3. U.S. Department of State. 1996 Patterns of Global Terrorism Report. Available from: URL: http://www.state.gov.
4. Carus WS. Bioterrorism and biocrimes: the illicit use of biological agents in the 20th Century [working paper]. Washington: Center for Counterproliferation Research, National Defense University; Aug 1998, revised Mar 1999.
5. Torok TJ, Tauxe RV, Wise RP, Livengood JR, Sokolow R, Mauvais S, et al. A large community outbreak of salmonellosis caused by intentional contamination of restaurant salad bars. JAMA 1997;278:389-95.
6. Departments of the Army, Navy, and Air Force. NATO Handbook on the Medical Aspects of NBC Defensive Operations. Washington: The Department; 1996.
7. Spertzel RO, Wannemacher RW, Patrick WC, Linden CD, Franz DR. Technical ramifications of inclusion of toxins in the chemical weapons convention (CWC). Technical report no. MR-43-92-1. Fort Detrick (MD): U.S. Army Medical Research Institute of Infectious Diseases; 1992.
8. World Health Organization Group of Consultants. Health aspects of chemical and biological weapons. Geneva: The Organization; 1970.
9. Department of the Army. U.S. army activity in the U.S. biological warfare programs. Vol II. Publication DTIC B193427L. Washington: The Department; 1977.
10. Vorobjev AA, Cherkassey BL, Stepanov AV, Kyuregyan AA, Fjedorov YM. Key problems of controlling especially dangerous infections. In: Proceedings of the International Symposium of Severe Infectious Diseases: Epidemiology, Express-Diagnostics and Prevention; 1997 Jun 16-20; Kirov, Russia. State Scientific Institution, Volgo-Vyatsky Center of Applied Biotechnology; 1997.
11. Alibek K, Handelman S. Biohazard. Random House. New York, NY; 1999.
12. Henderson DA. The looming threat of bioterrorism. Science 1999;283:1279-82.
13. Manchee RJ, Stewart WDP. The decontamination of Gruinard Island. Chemistry in Britain 1988;690-1.
14. The Holy Bible, new international version. Exodus Chapter 9. Indianapolis and Grand Rapids: B.B. Kirkbride Bible Company, Inc. and The Zondervan Corporation; 1978.
15. Centers for Disease Control and Prevention. Summary of notifiable diseases, 1945-1994. MMWR Morb Mortal Wkly Rep 1994;43:70-8.
16. Franz DR, Jahrling PB, Friedlander AM, McClain DJ, Hoover DL, Byrne WR, et al. Clinical recognition and management of patients exposed to biological warfare agents. JAMA 1997;278:399-411.
17. Centers for Disease Control and Prevention. Bioterrorism alleging use of anthrax and interim guidelines for management—United States, 1998. MMWR Morb Mortal Wkly Rep 1999;48:69-74.
18. Campbell GL, Hughes JM. Plague in India: a new warning from an old nemesis. Ann Intern Med 1995;122:151-3.
19. McNally RE, Morrison MB, Berndt JE, Fisher JE, Bo'Berry JT, Puckett V, et al. Effectiveness of medical defense interventions against predicted battlefield levels of botulinum toxin A. Joppa (MD): Science Applications International Corporation; 1994.
